# Evidence of reduced treatment adherence among HIV infected paediatric and adolescent populations in Nairobi at the onset of the UNAIDS Universal Test and Treat Program

**DOI:** 10.1186/s13104-018-3205-0

**Published:** 2018-02-17

**Authors:** Joseph Kabogo, Erastus Muniu, Fred Wamunyokoli, Rachel Musoke, Elijah Songok

**Affiliations:** 1 Jomo Kenyatta University of Agriculture and Technology (JKUAT), Juja, Kenya; 2 Department of Paediatrics, University of Nairobi, Nairobi, Kenya; 3 Kenya Medical Research Institute (KEMRI), Mbagathi Road, Nairobi, Kenya; 4 Centre for Public Health Research (CPHR), Kenya Medical Research Institute (KEMRI), Mbagathi Road, Nairobi, Kenya; 5 Department of Biochemistry, Jomo Kenyatta University of Agriculture and Technology (JKUAT), Juja, Kenya

**Keywords:** Universal test and treat, 90-90-90, HIV, Children, Antiretroviral therapy, Adherence, Virologic failure, Kenya

## Abstract

**Objective:**

We conducted a retrospective cohort study to evaluate the efficacy of the World Health Organization (WHO) “Universal Test and Treat” (UTT) policy, initiated in Kenya in September 2016. Under this policy, every human immunodeficiency virus (HIV)-infected person should be initiated on antiretroviral therapy (ART). We compared intra- and inter-group viral suppression and ART adherence rates for pre-UTT (initiated on ART in March–August 2016) and UTT groups (initiated in September 2016). The study was conducted in a community outreach Program in Nairobi with 3500 HIV-infected children enrolled.

**Results:**

122 children and adolescents were initiated on first-line ART pre-UTT, and 197 during the UTT period. The 6 month viral suppression rate was 79.7% pre-UTT versus 76.6% UTT (*P* < 0.05). Suboptimal adherence was higher in the UTT than pre-UTT period (88 of 197, 44.7% and 44 of 122, 34%; *P* < 0.001). The decrease in adherence was greater among orphans (91.7% pre-UTT and 87.2% UTT, *P* = 0.001) and children 11–18 years. Our results show that successful implementation of the UTT policy in Africa is challenged by an increased risk of suboptimal adherence. There is a need to develop extra strategies to support adherence, especially among orphans and teenagers.

**Electronic supplementary material:**

The online version of this article (10.1186/s13104-018-3205-0) contains supplementary material, which is available to authorized users.

## Introduction

Sub-Saharan Africa accounts for 71% of all Human Immunodeficiency Virus (HIV)-infected individuals, and 91% of all HIV-infected children below the age of 15 years [1]. In Kenya, there are approximately 190,000 HIV-infected children and adolescents, of whom only 38% are on antiretroviral therapy (ART) [2]. In 2014, the Joint United Nations Programme on HIV and AIDS (UNAIDS) launched the three 90-90-90 targets for 2020 as a major step towards eliminating the AIDS epidemic [3]. Related to this, the World Health Organization (WHO) directed that from the 1st of September 2016, every HIV-positive person should be initiated on ART immediately, regardless of their CD4+ T cell count [4]. This is commonly called the “Universal Test and Treat” (UTT) strategy. By availing ART to HIV-infected people and expanding prevention choices to the uninfected, 21 million AIDS-related deaths and 28 million new infections can be prevented by 2030 [5].

The 90-90-90 targets recognize and utilize ART as a life-saving treatment [3, 5, 6], a transmission prevention measure [5, 7] and a human right [5, 8]. Target one is successfully diagnosing 90% of all HIV-positive people. In target two, 90% of those diagnosed will be started on ART, and target three entails achieving viral suppression for 90% of those on ART. While allowing for serial 10% losses at each subsequent step, the implementation of these targets should result in 73% of all HIV-infected individuals achieving viral suppression [3, 5].

We conducted a retrospective cohort study whose objective was to determine the impact of the UTT policy on viral suppression and ART adherence rates among paediatric and adolescent populations in Nairobi, Kenya. A secondary objective was to determine the risk factors for virologic failure and suboptimal ART adherence.

## Main text

### Study site and methods

#### Study design and population

The Lea Toto Program (LTP) is a multi-Centre community outreach program in Nairobi, Kenya that cares for 3500 HIV-infected children and adolescents, aged 1–18 years old. These individuals live in eight low-income suburbs of Nairobi: Dagoretti, Dandora, Kangemi, Kariobangi, Kawangware, Kibera, Mukuru, and Zimmerman. The LTP Centres provide free ART drugs, CD4+ T-lymphocyte cell count monitoring, viral load testing, clinical management of opportunistic infections, and counselling.

This was a retrospective cohort study, which included all eligible participants going by inclusion criteria [9]. Two separate groups of patients were studied: the first group was the pre-UTT group, who began ART in any of the 6 months before the adoption of the UTT guidelines (March to August 2016). The second group was the UTT group: patients who began ART in September 2016, the UTT launch month. The data of interest for both groups was viral suppression and ART adherence rates 6 months after ART initiation. We conducted intra- and inter-group comparisons for the pre-UTT and UTT groups.

The retrospective cohort included 122 individuals in the pre-UTT group and 197 in the UTT group who were eligible going by the inclusion criteria: any children and adolescents who began ART during one of the two periods of interest (March-August 2016 or September 2016), and had been followed up for at least 6 months. Among the UTT group of 197 children, 108 children had tested positive in the pre-UTT period, but had not qualified for ART under the old WHO guidelines; either because they were above 10 years of age, or their CD4+ T-cell count was above 500 cells/µL [4]. Instead of ART, they had been on Co-trimoxazole prophylaxis prior to September 2016.

#### Data collection and analysis

Data was collected from patient files, and analysed using SPSS version 22. The WHO threshold of 1000 HIV-1 RNA copies/mL, was used to determine treatment success or virologic failure after 6 months of treatment [4]. Percentages, medians, and interquartile ranges (IQRs) were calculated. *P* values were determined using the Student’s T test for normally distributed data and the Mann–Whitney U test for skewed data. Univariate and multivariate Cox proportional hazards regression analysis was used to establish the effect of different variables on virologic failure. A threshold of *P* < 0.05 for statistical significance was set.

#### Adherence scores

To measure adherence rates, four parameters were used [10]: firstly, the pill counts done by the Clinicians during the monthly clinic visits; secondly, the pill counts done by the LTP community health workers (CHWs) during unannounced home visits, with the latter serving as verification of the former; thirdly punctuality in attending clinic sessions, with a maximum allowance of 2 days late; fourthly, undergoing the required blood tests on schedule. Each parameter contributed 25% to the overall score. Within each parameter, full compliance was scored as 25%, partial compliance was scored as 12.5%, and zero compliance was scored as 0%. Any overall score between 95 and 100% was defined as optimal, while a score below 95% was defined as suboptimal [11–13].

#### Ethical approval and consent to participate

The study was approved and cleared by the Kenya Medical Research Institute Scientific and Ethics Review Board, as well as the Nyumbani Medical Board (NMB); the NMB oversees all research and treatment operations in the LTP. All personal identifiers were removed by the researchers upon copying the data from the patient files. This was a retrospective cohort non-interventional study founded on post hoc analysis of data already present in patients’ files, and collected only for clinical indications [11]. Therefore, no written informed consent was asked of the patients.

### Results

#### Pre-UTT and UTT viral suppression intra-group comparisons

**Fig. 1:**
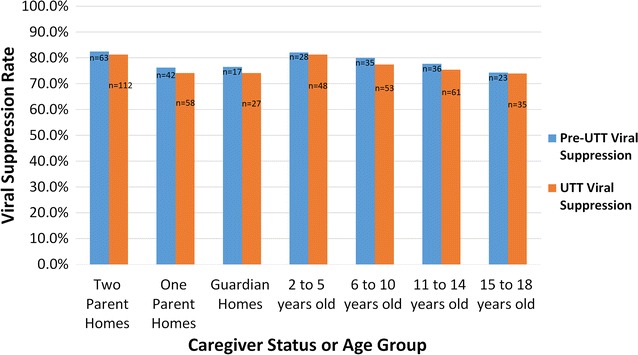
Viral Suppression rates stratified by caregiver status and age group for the 6 months before (pre-UTT) and after (UTT) the adoption of the Universal Test and Treat (UTT) strategy in a paediatric and adolescent population in Nairobi, Kenya

The baseline characteristics of the overall cohort, the pre-UTT group and the UTT group are shown in Table 1. We compared intra-group viral suppression rates within the pre-UTT group based on number of parents in the home and age group (Fig. 1). Two parent homes: 82.5%; one-parent homes: 76.2%, and guardian homes: 76.5%. For the comparison of two-parent homes vs. one-parent homes, *P* = 0.04; for two parent homes vs. guardian homes, *P* = 0.02. Age group comparisons: 2 to 5 years: 82.1%; 6–10 years: 80.0%; 11–14 years: 77.7%; 15–18 years: 73.9%. For the 2–10 years group vs. the 11–18 years group, *P* = 0.01. The UTT intra-group viral suppression rates are also shown in Fig. 1.

**Table 1:** Socio-demographic and clinical characteristics of the patients at baseline

Variable at baseline	All patients, n = 319	Pre-UTT patients, n = 122	UTT patients, n = 197
Age at baseline (years): median (IQR)	7.3 (3.0–11.2)	7.4 (3.6–11.2)	7.2 (2.1–11.0)
Gender			
Male	164 (51.4%)	63 (51.6%)	101 (51.3%)
Female	155 (48.6%)	59 (48.4%)	96 (48.7%)
Primary caregiver			
Both parents	100 (31.3%)	42 (34.5%)	58 (29.4%)
One parent	175 (54.9%)	63 (51.6%)	112 (56.9%)
Guardian	44 (13.8%)	17 (13.9%)	27 (13.7%)
HIV-type infection			
HIV-1	308 (96.5%)	118 (96.7%)	190 (96.5%)
HIV-2	4 (1.3%)	1 (0.1%)	3 (1.5%)
HIV-1 and HIV-2	7 (2.2%)	3 (3.2%)	4 (2.0%)
HIV-1 RNA, log_10_ copies/mL: median (IQR)	4.80 (4.07–5.49)	4.82 (4.10–5.48)	4.72 (3.85–5.52)
CD4+ T-cell count			
Below 200 cells/µL	58 (18.3%)	21 (16.8%)	37 (18.8%)
200–349 cells/µL	70 (21.7%)	29 (23.4%)	41 (20.8%)
350–499 cells/µL	94 (29.6%)	34 (28.1%)	60 (30.5%)
500 or more cells/µL	97 (30.4%)	38 (31.7%)	59 (29.9%)
CD4+ T-cell count, cells/µL: median (IQR)	432 (258–713)	423 (266–741)	466 (243–695)
WHO clinical stage			
Early stage(1 and 2)	122 (38.2%)	45 (36.7%)	77 (39.1%)
Advanced stage (3 and 4)	197 (61.8%)	77 (63.3%)	120 (60.9%)
1st line ART regimen			
AZT/3TC/NVP	139 (43.7%)	54 (44.3%)	85 (43.1%)
AZT/3TC/EFV	41 (12.9%)	17 (13.7%)	24 (12.2%)
ABC/3TC/NVP	58 (18.2%)	20 (16.5%)	38 (19.3%)
ABC/3TC/EFV	43 (13.3%)	18 (14.6%)	25 (12.7%)
TDF/3TC/EFV	24 (7.6%)	10 (8.1%)	14 (7.1%)
ABC/3TC/LPV/r	9 (2.8%)	2 (1.7%)	7 (3.6%)
AZT/3TC/LPV/r	5 (1.5%)	1 (1.1%)	4 (2.0%)

*UTT* Universal Test and Treat, *IQR* interquartile range, *WHO* World Health Organization, *ART* antiretroviral therapy, *ABC* abacavir, *3TC* lamivudine, *AZT* zidovudine, *TDF* tenofovir, *NVP* nevirapine, *EFV* efavirenz, *LPV/r* lopinavir/ritonavir

#### Pre-UTT and UTT viral suppression inter-group comparisons

The viral suppression rates were 79.7% pre-UTT vs. 76.6% UTT; *P* = 0.007. Significant reductions were observed for children under the care of guardians (76.5% pre-UTT vs. 74.1% UTT, *P* = 0.02) and the 11–14 years group (77.7% pre-UTT vs. 75.4% UTT *P* = 0.03). The 15–18 years group reported the lowest viral suppression rates among all age groups, both in the pre-UTT and UTT periods (73.9 and 74.3%, *P* = 0.35) (Fig. 1).

#### Risk factors for below-target viral suppression

The variables considered as potential risk factors were adherence level, caregiver status, gender, and age group. The treatment success sub-group was compared to the virologic failure sub-group within the pre-UTT and UTT groups (Table 2). These variables qualified as risk factors when three conditions were met: the Cox proportional hazard ratio (HR) had to be greater than 1.96, both the lower and upper limits of the 95% confidence interval (CI) had to be greater than 1, and the *P* value had to be less than 0.05 [14]. Suboptimal adherence was the only predictive risk factor: children with suboptimal adherence in the pre-UTT group were 5.3 times more likely to develop virologic failure when compared to individuals in the same group with optimal adherence [univariate hazard ratio (HR) = 5.32, 95% confidence interval (CI) 2.75–10.29, *P* value of 0.0006]. The likelihood of treatment failure increased to 14.6 times after implementation of UTT (univariate HR = 14.63, 95% CI 3.18–104.38, *P* value of 0.003), a 2.8-fold increase (Table 2).

**Table 2:** Univariate and multivariate Cox proportional hazards regression (CPHR) of possible risk factors for below-target viral suppression

Characteristic	Univariate analysis			Multivariate analysis		
	Hazard ratio	*P* value	95% confidence interval	Hazard ratio	*P* value	95% confidence interval
Pre-UTT period (March to August 2016): risk factors for below-target viral suppression						
Optimal adherence to ART	1.0			1.0		
Suboptimal adherence to ART	*5.32*	*0.0006*	*2.75–10.29*	*2.29*	*0.007*	*1.21–3.39*
UTT period (September 2016 to February 2017): risk factors for below-target viral suppression						
Optimal adherence to ART	1.0			1.0		
Suboptimal adherence to ART	*14.63*	*0.003*	*3.18–104.38*	*6.24*	*0.01*	*2.63–17.82*
Pre-UTT period (March to August 2016): risk factors for suboptimal adherence						
Two parent homes	1.0			1.0		
One parent homes	1.13	0.42	0.84–1.53	1.07	0.67	0.79–1.45
Guardian homes	1.81	0.08	0.97–3.77	1.09	0.60	2.80–1.48
2–5 years old	1.0			1.0		
6–10 years old	0.50	0.03	0.34–0.73	0.88	0.19	0.72–1.07
11–14 years old	1.45	0.01	0.81–2.69	0.84	0.042	0.71–0.99
15–18 years old	0.40	0.05	0.27–0.60	0.73	0.02	0.56–0.89
Female gender	1.0			1.0		
Male gender	0.66	0.02	0.53–0.82	0.81	0.03	0.73–0.91
UTT period risk factors for suboptimal adherence						
Two parent homes	1.0			1.0		
One parent homes	1.08	0.03	1.44–9.90	1.11	0.90	0.22–5.64
Guardian homes	*2.27*	*0.041*	*1.12–6.72*	1.13	0.80	2.19–6.62
1–5 years old	1.0			1.0		
6–10 years old	1.17	0.73	0.48–2.85	1.11	0.90	0.22–5.64
11–14 years old	*2.16*	*0.018*	*1.03–5.73*	1.02	0.20	0.07–14.47
15–18 years old	1.84	0.95	0.33–3.28	1.20	0.27	0.01–3.59
Female gender	1.0			1.0		
Male gender	0.73	0.56	0.26–2.07	1.02	0.20	0.07–14.47

The variables considered were adherence to ART, caregiver status, and gender. Another CPHR analysis was conducted to determine risk factors for suboptimal adherence to ART. The variables considered were caregiver status, age group, and gender. The CPHR analyses were done for both the Pre-UTT period (September 2016 to February 2017) and the UTT period (March to August 2016)

Italic values indicate significance of *P* value (*P* < 0.05)

#### Risk factors for suboptimal adherence

Children with suboptimal adherence increased from 36% (44 of 122) in the pre-UTT group to 44.7% (88 of 197) in the UTT era (*P* < 0.001). The complete adherence dataset is found in Additional file 1: Appendix S1. The variables considered as potential risk factors for suboptimal adherence were caregiver status, age group, and gender. For the pre-UTT group, none of these variables met all three conditions for predictive Cox hazard ratios [14]. However in the UTT period, both guardian caregiver status and being 11–14 years old were predictive of suboptimal adherence: UTT guardian homes: univariate HR = 2.27, 95% CI 1.12–6.72, *P* = 0.041); children aged 11–14 years: Univariate HR = 2.16, 95% CI 1.03–5.73, *P* = 0.018 (Table 2).

### Discussion

Our study shows a significant fall in viral suppression rates among an HIV-infected paediatric and adolescent population in Nairobi on the transition from the previous WHO treatment guidelines to the UTT policy. This was directly correlated with a fall in adherence rates. Children with suboptimal adherence were 5.3 times more likely to fail ART pre-UTT, and 14.6 times more likely to fail in the UTT period, representing a 2.8-fold increase in the rate of virologic failure. Previous studies have proved the direct link between poor adherence to ART and increased risk of treatment failure [15–17]. This is even more concerning in the light of UTT ART scale-up for the 22 million people who were not on ART before September 2016 [18, 19]. Prior to UTT implementation, patients received thorough ART counselling before being initiated on treatment. In the “test and start” UTT dispensation, patients receive ART counselling concurrent with treatment [4]. Therefore, patients in the UTT period are more likely to have a poorer understanding of the importance of taking their medicine at specific times of the day, every day. This may result in lower viral suppression and adherence rates.

Adolescent populations have been shown in previous studies to be the least adherent to ART. A recent national survey in Kenya found higher levels of suboptimal adherence among persons aged 15–29 [20]. This is in conformity with data from across sub-Saharan Africa which found that ART adherence by pre-teenagers and teenagers is influenced by disclosure [21, 22], socioeconomic status [23], treatment fatigue [24], fear of stigmatization by peers [25], and vigilance by the primary caregivers [12, 13]. In the UTT period, many more pre-teenagers and teenagers who had not qualified for ART under the old WHO guidelines were initiated on ART. This transition may have amplified the above factors.

Children under the care of guardians in our study showed decreased adherence rates and a corresponding increase in treatment failure upon the transition to UTT. This is in line with previous studies which show that HIV-infected orphans being cared for by guardians are at greater risk of poor adherence and treatment failure [26–28]. In Zambia, higher adherence levels were observed where the child’s mother was the primary caregiver and improved further if the child had multiple caregivers [13]. In Rwanda, double orphans under the care of guardians or siblings had the lowest adherence rates to ART [29]. It has been observed that non-biological caregivers are less motivated to monitor their children’s ART ingestion as compared to biological parents [30]. This may be more exacerbated in an urban slum setting, such as the LTP cohort in our study, where individuals live on less than 5 US dollars daily.

Our observations show an increase in suboptimal adherence and a corresponding increase in treatment failure rates among a paediatric and adolescent population in Nairobi, Kenya at the onset of implementation of the WHO UTT Program in September 2016. Though there is overwhelming scientific evidence that starting ART as soon as possible has profound advantages, there is need to develop extra strategies to vigorously support adherence especially among orphaned children and teenagers.

## Limitations

The main limitation of the study was that it was a retrospective cohort study with only 6 months of follow up. A time series analysis over 12–24 months would likely reveal more. Secondly, although the total number of children in the LTP is large (3500), only about 20–30 children were initiated on ART monthly in the pre-UTT period. Thus, we were able to include only 122 children in the pre-UTT group and 197 in the UTT group who met inclusion criteria. A larger sample size would have powered the study.

## Additional file

**Additional file 1: Appendix S1.** A PDF file with a detailed dataset for intra- and inter-group Adherence rates, before the adoption of Universal test and Treat (UTT) and after UTT adoption; found at https://doi.org/10.6084/m9.figshare.5760219.v1.

## Abbreviations

UTT: Universal Test and Treat
UNAIDS: United Nations Programme on HIV and AIDS
WHO: World Health Organization
LTP: Lea Toto Programme
AIDS: acquired immune deficiency syndrome
HIV: human immunodeficiency virus
ART: antiretroviral therapy
CHWs: community health workers
HR: hazard ratio
CI: confidence interval
IQR: interquartile range
ABC: abacavir
3TC: lamivudine
AZT: zidovudine
TDF: tenofovir
NVP: nevirapine
EFV: efavirenz
LPV/r: lopinavir/ritonavir
