# Supplementary material for: Evidence of reduced treatment adherence among HIV infected paediatric and adolescent populations in Nairobi at the onset of the UNAIDS Universal Test and Treat Program
Source: BMC Res Notes. 2018 Feb 17;11:134. doi: 10.1186/s13104-018-3205-0 (PMC5816400; doi:10.1186/s13104-018-3205-0)
Supplement: Supplementary file 1 — Additional file 1: Appendix S1. A PDF file with a detailed dataset for intra- and inter-group Adherence rates, before the adoption of Universal test and Treat (UTT) and after UTT adoption; found at https://doi.org/10.6084/m9.figshare.5760219.v1. [file 13104_2018_3205_MOESM1_ESM.pdf]

Supplementary data: Additional File 1.

https://doi.org/10.6084/m9.figshare.5760219.v1

Evidence of reduced treatment adherence among HIV infected paediatric and adolescent populations in Nairobi at the onset of the UNAIDS Universal Test and Treat Program.

Joseph Kabogo[1,3], Erastus Muniu[4], Fred Wamunyokoli[5], Rachel Musoke[2] and Elijah Songok[3*]

[1] Jomo Kenyatta University of Agriculture and Technology (JKUAT), Juja, Kenya.
[2] Department of Paediatrics, University of Nairobi, Nairobi, Kenya.
[3] Kenya Medical Research Institute (KEMRI), Mbagathi Road, Nairobi, Kenya.
[4] Centre for Public Health Research (CPHR), Kenya Medical Research Institute (KEMRI), Mbagathi Road, Nairobi, Kenya.
[5] Department of Biochemistry, Jomo Kenyatta University of Agriculture and Technology (JKUAT), Juja, Kenya.

Contact Emails:
Joseph Mbugua Kabogo: Josephkabogo@gmail.com
Erastus Muniu: Emuniu@kemri.org
Fred Wamunyokoli: fwamunyokoli@gmail.com
Rachel Musoke: rachelnamu@africaonline.co.ke

*Corresponding author:
Elijah Songok, PhD
Kenya Medical Research Institute
Mbagathi Road, Nairobi
Email: Elijah.Songok@umanitoba.ca; Esongok@kemri.org

Pre-UTT and UTT groups Adherence rates intra-group comparisons.

Overall adherence to ART for the pre-UTT group was 94.0% (n = 122). The two parent homes rate: 96.1% (n = 63); one parent homes rate: 94.3% (n = 42); guardian homes rate: 91.7% (n = 17) (**Supplementary Figure 1**). Two parent homes rate and the guardian homes rate comparison: *P* = 0.016. Age group comparisons, pre-UTT group: the 2 to 5 years old group: 97.1% (n = 28); the 6 to 10 years old group: 94.9% (n = 35); the 11 to 14 years old group: 91.3% (n = 37); the 15 to 18 years old age group: 93.5% (n = 22). The 2 to 10 years group vs the 11 to 18 years group: *P* = 0.04 (**Supplementary Figure 1**).

For the UTT group, the overall adherence to ART rate was 90.8% UTT (n = 197). Two parent homes (93.5%) and the guardian homes (87.2%), with *P* = 0.008. For the comparison of the 2 to 10 years group vs the 11 to 18 years group, *P* = 0.01. Gender was tested as a variable but did not show a statistically significant difference.

Pre-UTT and UTT groups Adherence rates inter-group comparisons.

Pre-UTT and UTT inter-group comparisons of ART adherence: 94.0% pre-UTT (n = 122) and 90.8% UTT (n = 197), with *P* = 0.006). For two parent homes, the adherence rate of 96.1% (n = 63) pre-UTT and 93.5% (n = 112) UTT (*P* = 0.005); One parent homes rates: 94.3% (n = 42) pre-UTT and 91.8% (n = 58) UTT (*P* = 0.018); guardian homes rates: 91.7% (n = 17) pre-UTT and 87.2% (n = 27) UTT (*P* = 0.001) (**Supplementary Figure 1**).

For the 2 to 5 years old group, the adherence rate was 97.1% (n = 28) pre-UTT and 95.9% (n = 50) UTT (*P* = 0.13); the 6 to 10 years old group: 94.9% (n = 35) pre-UTT and 93.6% (n = 48) UTT (*P* = 0.12); the 11 to 14 years old group: 91.3% (n = 37) pre-UTT and 88.7% (n = 54) UTT (*P* = 0.016). The 15 to 18 years old age group had rates of 93.5% (n = 22) pre-UTT and 90.8% (n = 45) UTT (*P* = 0.014) (**Supplementary Figure 1**).

The change in suboptimal adherence in the various categories between the pre-UTT and UTT period was examined**.** Overall, all categories experienced a reduction in adherence level on transition to UTT, which was directly reflected in a reduction in viral suppression rates (**Figure 1 and Supplementary Figure 1**)**.** The child's family background significantly predicted adherence levels, with those raised in two parent families doing better than those of single parentage. Children raised by guardians had the lowest adherence levels (91.7 % Pre-UTT and 87.2% UTT, *P*=0.001). For age groups**,** pre-teens and early teens (ages 11-14 years) were the

most impacted on adherence after introduction of UTT with no improvement recorded on adolescents who showed the least adherence during both time periods (91.3 % Pre-UTT and 88.7% UTT, *P*=0.016).

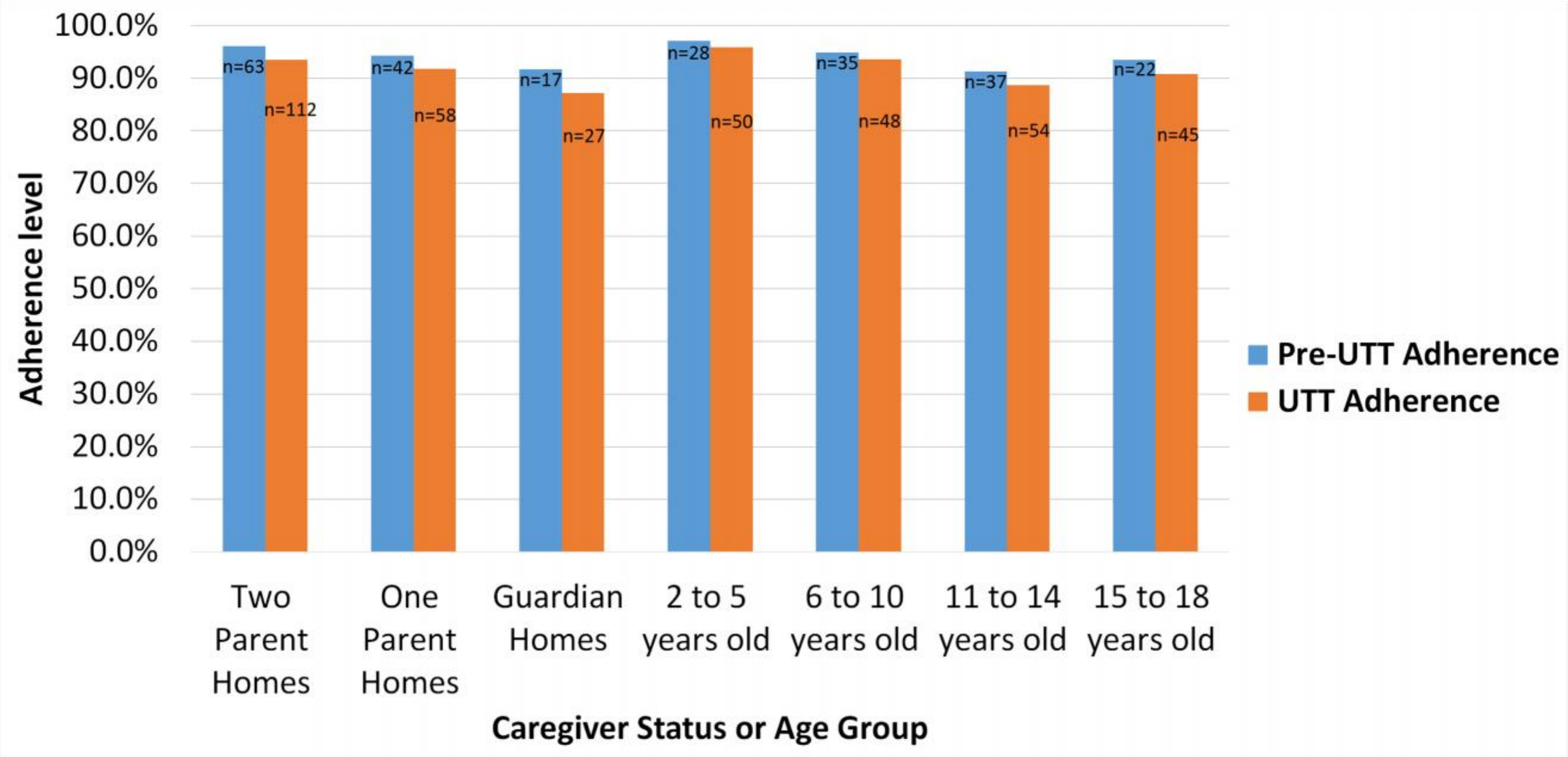

Supplementary Figure 1: Adherence levels stratified by Caregiver status and Age Group for the 6 months before (pre-UTT) and after (UTT) the adoption of the Universal Test and Treat Strategy (UTT) in a paediatric and adolescent population in Nairobi.
